# Participation of PLK1 and FOXM1 in the hyperplastic proliferation of pulmonary artery smooth muscle cells in pulmonary arterial hypertension

**DOI:** 10.1371/journal.pone.0221728

**Published:** 2019-08-22

**Authors:** Jamie L. Wilson, Lizhen Wang, Zeyu Zhang, Nicholas S. Hill, Peter Polgar

**Affiliations:** 1 Tupper Research Institute and Pulmonary, Critical Care, and Sleep Division, Tufts Medical Center, Boston, Massachusetts, United States of America; 2 Department of Pulmonary and Critical Care Medicine, Guangdong Second Provincial General Hospital, Guangdong Province, Guangzhou, China; 3 Postdoctoral Research Station of Clinical Medicine, The Third Xiangya Hospital, Central South University, Changsha, China; Stanford University, UNITED STATES

## Abstract

Vascular smooth muscle cells from the pulmonary arteries (HPASMC) of subjects with pulmonary arterial hypertension (PAH) exhibit hyperplastic growth. The PAH HPASMC display an increased sensitivity to fetal bovine serum (FBS) and undergo growth at a very low, 0.2%, FBS concentration. On the other hand, normal HPASMC (obtained from non-PAH donors) do not proliferate at low FBS (0.2%). A previous genomic study suggested that the nuclear factor, FOXM1 and the polo like kinase 1 (PLK1) are involved in promoting this hyperplastic growth of the PAH HPASMC. Here we find that limiting the action of FOXM1 or PLK1 not only restricts the hyperplastic proliferation of the PAH HPASMC but also modulates the FBS stimulated growth of normal HPASMC. The PAH HPASMC exhibit significantly elevated PLK1 and FOXM1 expression and decreased p27 (quiescence protein) levels compared to normal HPASMC. Regulation of the expression of FOXM1 and PLK1 is accompanied by the regulation of downstream expression of cell cycle components, Aurora B, cyclin B1 and cyclin D1. Expression of these cell cycle components is reversed by the knockdown of FOXM1 or PLK1 expression/activity. Furthermore, the knockdown of PLK1 expression lowers the protein level of FOXM1. On the other hand, inhibiting the action of FOXO1, a growth inhibitor, further increases the expression of FOXM1 in PAH HPASMC. Although PLK1 and FOXM1 clearly participate in PAH HPASMC hyperplasia, at this time it is not clear whether their increased activity is the primary driver of the hyperplastic behavior of the PAH HPASMC or merely a component of the pathway(s) leading to this response.

## Introduction

In a previous communications we showed that smooth muscle cells from the pulmonary artery of PAH subjects display abnormal behavior which manifests as hyperplastic growth [1] and dysregulated migration [2]. The hyperplastic growth phenomenon has also been demonstrated by others [3–5]. Regardless, these human pulmonary artery smooth muscle cells (HPASMC) isolated and cultured from subjects both with or without PAH retain their *in vivo* phenotype as illustrated by their expression of both alpha smooth muscle actin and H-caldesmon [1] and constriction in response to endothelin 1 [6]. The hyperplastic phenotype from subjects with PAH is characterized by continued growth under normally non-proliferative, non-growth stimulated cell culture conditions (1).

Our gene microarray communication on HPASMC from PAH and non-PAH pulmonary arteries suggested that several genes which are involved in cellular proliferation and cell cycle regulation are activated in the PAH derived HPASMC [7]. The oncogene FOXM1 and cell cycle regulator, polo-like kinase 1 (PLK1), were of particular note as possibly contributing to this hyperplastic growth as they were upregulated in PAH HPASMC [7].

FOXM1 has now been shown to regulate hypoxia-induced proliferation in normal HPASMC [8]. Additionally, in mice, a constitutively active FOXM1 transgene induced epithelial hyperplasia when expressed in lung epithelial cells [9]. Other studies have confirmed that FOXM1 is upregulated in pulmonary arteries from PAH patients and animals with experimental pulmonary hypertension. Inhibition of FOXM1 by genetic ablation or use of its pharmacological inhibitor, thiostrepton, indeed ameliorates experimental hypertension [10–12].

FOXM1 is a member of the forkhead box transcription factor family known to function in cell proliferation [13], cell cycle progression [14] and pulmonary vascular development [9, 15–18]. It regulates the expression of multiple genes participating in G1 to S and G2 to M progression including *PLK1*, *Aurora B*, multiple cyclin family members, and *p27*^*Kip1*^ (*p27*) [13, 19–21]. p27 (cyclin dependent kinase inhibitor 1B) is a kinase which disrupts the activation of multiple cyclin-cyclin dependent kinase complexes thus slowing down progression of the cell cycle. PLK1 is an essential regulator of the cell cycle. It is known to regulate exit and entrance of the cell into mitosis. In humans, PLK1 is often overexpressed in a broad spectrum of cancers and its overexpression correlates with prognosis [22, 23]. Blocking the expression or action of PLK1 can effectively inhibit the proliferation of tumor cells [24]. Little has been done to investigate PLK1’s participation in PAH.

Another transcription factor of interest in PAH is the forkhead box protein O1 (FOXO1). FOXO1 participates in the regulation of cell cycle progression [25]. It has been reported to regulate the cell cycle via induction of p27 and transcriptional repression of D-type cyclins [26, 27]. Savai et al. reported that expression of FOXO1 is down-regulated in human IPAH and experimental pulmonary hypertensive (PH) rodent lungs [28]. Evidence points to a link between PLK1 induced phosphorylation of FOXM1 and the inactivation of FOXO1 via its phosphorylation thus further promoting cell proliferation [29, 30]. This makes PLK1 an attractive therapeutic target for PAH therapy as its inhibition would potentially decrease detrimental FOXM1 activity while increasing beneficial FOXO1 action, consistent with the reported opposing roles of FOXM1 and FOXO1 in regulation of cell proliferation [31]. All this in sum suggests a balance of PLK1, FOXO1 and FOXM1 activity and expression as key to normal regulation of the cell cycle and cell proliferation in the HPASMC and a possible key to the dysregulation of growth of the PAH HPASMC.

Herein we investigate the expression and actions of FOXM1 and PLK1 and their effect on cell cycle regulatory components Aurora B, cyclin D1, cyclin B1 in relation to normal and hyperplastic growth of HPASMC. Our results illustrate that the actions and expression of FOXM1 and PLK1 are intimately interwoven and contribute to the regulation of Aurora B, cyclin B1 and cyclin D1 expression. Results are promising and raise the possibility that through manipulation of the expression and actions of PLK1 and FOXM1 the hyperplastic proliferation of HPASMC contributing to the obstruction of the vascular lumen can be alleviated or even normalized.

## Material and methods

### Chemicals and reagents

Pharmacological inhibitor, thiostrepton was purchased from Santa Cruz Biotechnology, Inc (Dallas, TX), volasertib (BI 6727) from Selleckchem (Houston, TX) and AS1842856 from Cayman Chemical (Ann Arbor, Michigan). Silencer Select siRNAs (targeting FOXM1, PLK1, FOXO1 and negative control), Lipofectamine RNAiMAX and Opti MEM were purchased from Thermo Fisher Scientific (Waltham, MA). MTT assay kits were purchased from ATCC (Manassas, VA) and BrdU assay kits were purchased from BioVision, Inc. (Milpitas, CA).

### Cell culture

Human pulmonary artery smooth muscle cells (HPASMC) derived from non-PAH, hereditary PAH (HPAH) and idiopathic PAH (IPAH) were isolated as described by Comhair et al. 2012 [32]. They were a generous gift from Drs. Erzurum and Comhair of the Cleveland Clinic (Cleveland, OH) and Dr. Marlene Rabinovitch of Stanford University under the Pulmonary Hypertension Breakthrough Initiative. Details of the cellular derivation can be found in a previous communication Yu et al. 2013 [33]. Briefly, the cells were isolated from elastic pulmonary arteries (>500-μm diameter) from explanted lungs of PAH patients and non-PAH donors. Cells were cultured in 15 mM HEPES buffered DMEM/F12 (50:50) media (Thermo Fisher Scientific, Cat # 11330032, Waltham, MA) containing 10% fetal bovine serum (Atlanta Biologicals, Cat # S115500, Lot # A17004, Flowery Branch, GA), and 2.5% Antibiotic-Antimycotic (Thermo Fisher Scientific Cat # 15240). Cells were passaged at 60–90% confluence by dissociation from plates with 0.05% trypsin and 0.53 mM EDTA. Primary cultures, passages 6–10, were used in herein. The smooth muscle cell phenotype of these cells was confirmed via immunostaining for alpha smooth muscle actin [1, 32]. All cell strains in this study and other published studies (not used directly here) have consistently shown that PAH HPASMC exhibit sizably increased proliferation, survival and anti-apoptosis in culture akin to their behavior in vivo ([1–5, 10, 12, 28, 34–39]. Specific information about the cell donors used in this study is shown in **S1 Table**.

The HPASMC were maintained at 0, 0.2 and 5% FBS concentrations as needed to examine expression and behavior under proliferative and non-proliferative conditions. While the PAH HPASMC were stimulated to growth at 0.2% FBS the non-PAH cells were not and required 5% FBS for robust growth.

### Real-time qPCR

RNA was extracted from HPASMC using TRIzol reagent (Thermo Fisher Scientific, Waltham, MA) according to manufacturer’s specifications. Real-time quantitative PCR was performed by generating cDNA from 200 ng total RNA using the High-Capacity cDNA Reverse Transcription Kit (Thermo Fisher Scientific, Waltham, MA) according to manufacturer’s specifications. Gene expression was determined by running samples on a QuantStudio 3 instrument using PowerUp SYBR Green Master Mix (Thermo Fisher Scientific, Waltham, MA) according to manufacturer’s specifications. Results are presented as relative expression normalized to 18S RNA and were calculated using the ΔΔCt method. Human primer sequences used are the following: FOXM1 For-AGCAGTCTCTTACCTTCC; FOXM1 Rev-CTGGCAGTCTCTGGATAA; PLK1 For-TGATGGCAGCCGTGACCTA; PLK1 Rev-GGCGGTATGTGCGGAAGT; 18S For-GTAACCCGTTGAACCCCATT; 18S Rev-CCATCCAATCGGTAGTAGCG.

### Western blot

Cells were lysed using RIPA buffer containing both Protease Inhibitor Cocktail and Phosphatase Inhibitor Cocktail 2 (Sigma-Aldrich, St Louis, MO). Cell lysates were clarified by centrifugation for 20 min at 4°C. The protein concentration of each cleared lysate was determined using bicinchoninic acid assay (BCA) (Thermo Fisher Scientific, Waltham, MA) according to manufacturer’s specifications. Equal amounts of protein were loaded and electrophoresed on 7% or 10% SDS-PAGE. Proteins were then transferred onto Immobilon-P 0.45 um PVDF membrane (EMD Millipore, Darmstadt, Germany) at 100 V for 1.5 hour. The PVDF membranes were treated for 1 hour with 5% powdered milk in TBS-T. After blocking, PVDF membranes were probed with one of the following antibodies overnight at at 4°C diluted in TBS-T with 5% BSA: anti-FOXM1 (1:1000, sc-502, Santa Cruz Biotechnology Inc, Dallas, TX), anti-PLK1 (1:1000, ab17056, Abcam, Cambridge, UK), anti-FOXO1 (1:500, 2880, Cell Signaling Technologies, Danvers, MA), anti-Aurora B (1:500, 3094, Cell Signaling Technologies, Danvers, MA), anti-cyclin B1 (1:500, 12231, Cell Signaling Technologies, Danvers, MA), anti-cyclin D1 (1:1000, 2978, Cell Signaling Technologies, Danvers, MA), anti- p27^Kip1^ (1:750, 2947, Cell Signaling Technologies, Danvers, MA) and anti-beta actin (1:1000, 3700, Cell Signaling Technologies, Danvers, MA). Membranes were washed again in TBS-T followed by 1 h incubation with the corresponding anti-rabbit or anti-mouse 2° Ab (7074, 7076, Cell Signaling, Danvers, MA) diluted 1:2000 TBS-T. Blots were then washed in TBS-T and developed using SuperSignal West Pico PLUS Chemiluminescent Substrate (Thermo Fisher Scientific, Waltham, MA) for 1 min before imaging on a Fluor Chem E system (Protein Simple, San Jose, CA). Densitometry on band intensities was performed using the NIH ImageJ image analysis software [40]. Protein expression was quantified using band intensity values (in arbitrary units), which were normalized to beta actin. The intensity of the western bands for FOXM1 and PLK1 was relatively low especially in the normal-HPASMC because the concentration of these proteins is low and was reaching the maximum loading level with regard to the sensitivity of the available antibodies in the HPASMC. Nevertheless, the bands as shown proved quantitatively reproducible in all experiments presented.

### Cell proliferation

The proliferation of HPASMC was determined with two assays, BrdU and MTT. Very similar results were obtained with either assay. One advantage of the MTT assay is that it also evaluates cell viability.

#### Asynchronous cell proliferation

HPASMC were seeded in 96 well culture plates at a density of 3,500 cells/well. After attachment the medium was immediately changed to respective FBS concentration of SMC medium with or without respective inhibitors or 0.01% DMSO. After 5 days, cell proliferation was measured using dimethyl thiazolyl diphenyl tetrazolium salt (MTT) or BrdU assay according to the manufacturer’s standard procedure (ATCC, Manassas, VA). The day before MTT measurements triplicate wells on the same plate were loaded with either 0.5X, 1X or 2X of the original number of cells seeded so that correlations between the OD and cell number could be determined. OD measurements were made at 560 or 450 nm on spectrophotometer. Simultaneous experiments using cell counts and the MTT assay confirmed uniform cell proliferation results with the two assays. All treatment groups were done in at least triplicate wells.

#### Synchronous cell proliferation

HPASMC were seeded in 96 well culture plates at a density of 3,500 cells/well. After attachment the medium in each well was changed to serum free medium for 24 h. The cell culture wells were then pretreated with respective inhibitors or 0.01% DMSO for 1 h before the addition of culture medium containing FBS for 48 h. BrdU was added to each well during the last 18 h. The culture plate was processed according to the manufacturer’s standard procedure (BioVision, Inc., Milpitas, CA). OD was then measured at 450 nm after 10 min in stop solution. All treatment groups were done in at least triplicate wells.

### Transfection with siRNA

Validated Silencer Select pre-designed siRNAs targeting FOXM1, PLK1, FOXO1 and negative control siRNAs (60 pmol in 6 well plates or 2.5 pmol in 96 well plates) were transfected into HPASMC using Lipofectamine RNAiMAX Transfection Reagent (Thermo Fisher Scientific) for 48 h according to the manufacturer’s recommendations. The knockdown efficiency was determined by western blot analysis.

### Statistics

Statistical significance between the means of two groups was determined by Student’s t-test and comparisons between multiple groups was determined by one-way ANOVA with Dunnett’s post hoc test. P values less than 0.05 were considered significant. All statistical analyses were performed using Prism 8.2.0 (GraphPad Software, La Jolla, CA). The number of n refers to the number of biological replicates (each donor cell) used in experiments.

## Results

### Regulation of non-PAH and PAH HPASMC proliferation through FOXM1 and PLK1 action

**Fig 1:**
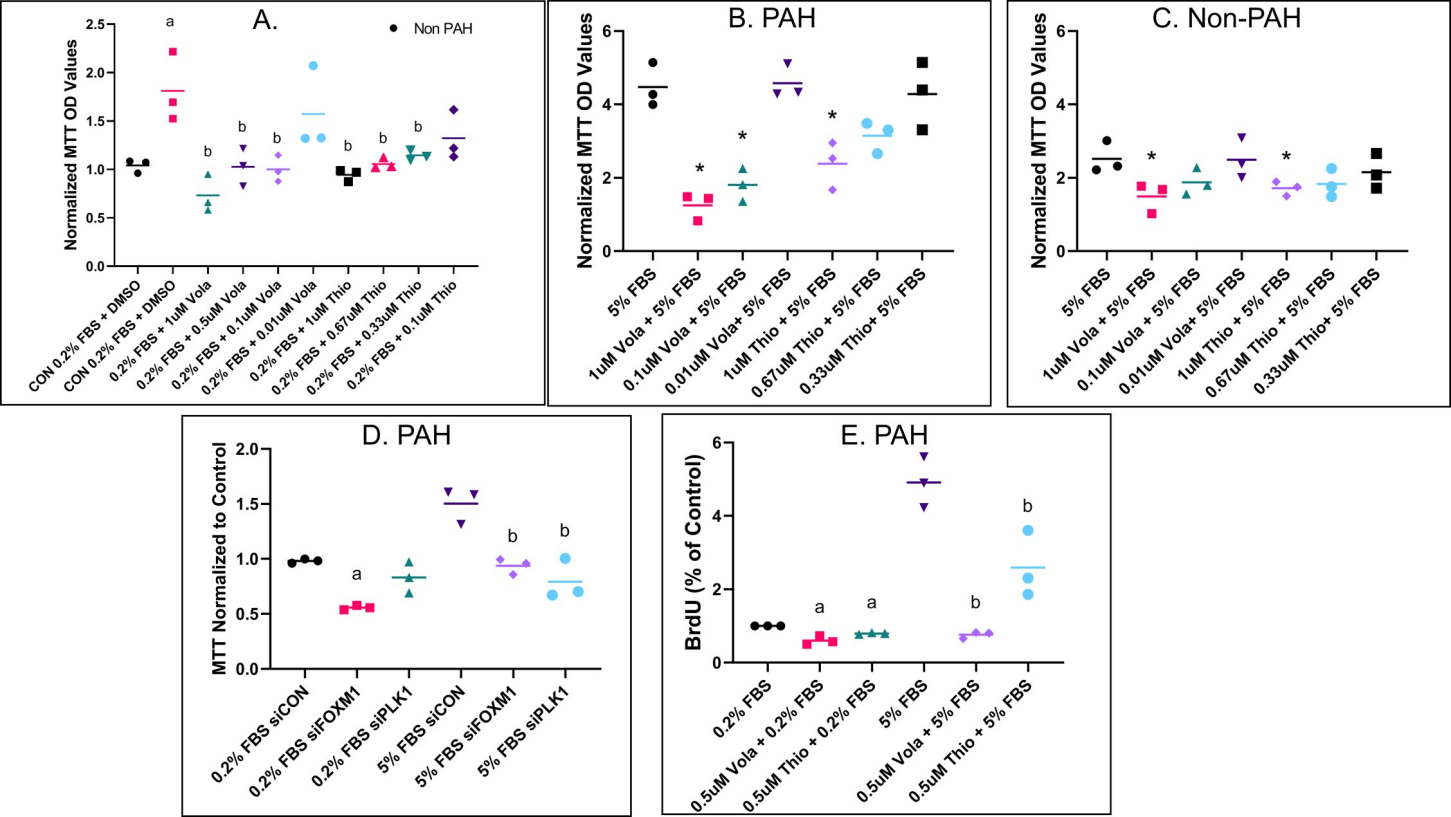
Regulation of HPASMC proliferation via FOXM1 and PLK1 inhibition. (A) Scatter plot showing non-PAH (black circles only) and PAH HPASMC proliferation in low serum (0.2% FBS) after five days in the presence or absence (DMSO) of PLK1 inhibitor, volasertib (vola) or FOXM1 inhibitor, thiostrepton (thio). MTT OD values were normalized to cells in 0% FBS medium. a = p < 0.05 vs non-PAH 0.2% FBS; b = p < 0.05 vs PAH 0.2% FBS. (B) Scatter plot showing PAH HPASMC proliferation in high serum (5% FBS) after five days in the presence or absence (DMSO) of PLK1 inhibitor, volasertib (vola) or FOXM1 inhibitor, thiostrepton (thio). MTT OD values were normalized to cells in 0% FBS medium. * = p < 0.05 vs 5% FBS. (C) Scatter plot showing non-PAH HPASMC proliferation in high serum (5% FBS) after five days in the presence or absence (DMSO) of PLK1 inhibitor, volasertib (vola) or FOXM1 inhibitor, thiostrepton (thio). MTT OD values were normalized to cells in 0% FBS medium. * = p < 0.05 vs 5% FBS. (D) Scatter plot showing PAH HPASMC proliferation in low serum and high serum after five days in the presence of siFOXM1, siPLK1 or control siRNA. MTT OD values were normalized to 0.2% FBS siCON. a = p < 0.05 vs 0.2% FBS siCON; b = p < 0.05 vs 5% FBS siCON. (E) Scatter plot showing PAH HPASMC proliferation after five days via BrdU assay. BrdU OD values were normalized to 0.2% FBS control. a = p < 0.05 vs 0.2% FBS; b = p < 0.05 vs 5% FBS. Experiments were done in triplicate with n = 3 non-PAH and n = 3 PAH cells. Line in scatter plots represents the mean.

As described previously, PAH HPASMC proliferate under normally quiescent cell culture condition (0.2% FBS concentration) [1]. **Fig 1A** illustrates that PAH HPASMC indeed proliferate under low serum conditions, while normal control HPASMC do not. Furthermore, inhibitors of PLK1 and FOXM1 action significantly reduced the PAH HPASMC proliferation in a dose-dependent manner (**Fig 1A**). Cell proliferation of both normal and PAH HPASMC is also shown at a much higher, 5% FBS, concentration in **Fig 1B and 1C**. At this much higher FBS concentration proliferation of both normal and PAH HPASMC was also markedly reduced by inhibiting the action of either FOXM1 or PLK1 (**Fig 1B and 1C)**. siRNA knockdown of PLK1 or FOXM1 similarly reduced proliferation in PAH HPASMC under both low (0.2%) and high serum (5%) concentrations as shown in **Fig 1D**. To confirm the growth results obtained with use of the MTT assay, we additionally measured cell proliferation under the same conditions using BrdU incorporation and obtained results which mirrored those obtained with the MTT assay (**Fig 1E**).

**Fig 2:**
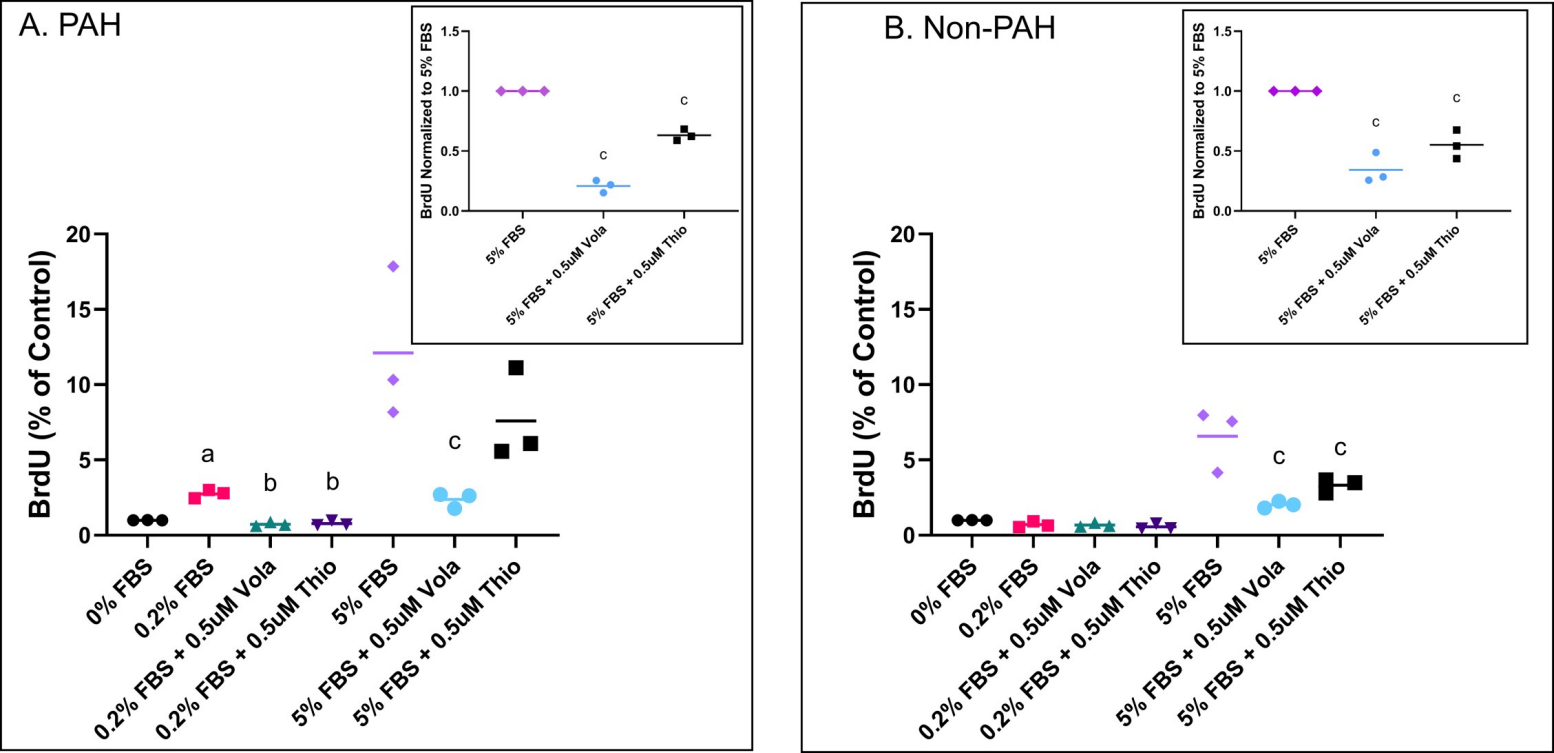
Regulation of HPASMC DNA synthesis via FOXM1 and PLK1 inhibition. PAH (A) and non-PAH (B) HPASMC were assayed for BrdU incorporation after pretreatment with PLK1 inhibitor, volasertib (vola), or FOXM1 inhibitor, thiostrepton (thio) for 1 h and stimulated with either vehicle, 0.2% FBS or 5% FBS culture medium for 48 h. BrdU OD values were normalized to 0% FBS control. Insets contain BrdU OD values that were normalized to 5% FBS for comparison minus biological variation. a = p < 0.05 vs 0% FBS; b = p < 0.05 vs 0.2% FBS; c = p < 0.05 vs 5% FBS. Experiments were done in triplicate with n = 3 non-PAH and n = 3 PAH cells. Line in scatter plots represents the mean.

Since these experiments measured proliferation during asynchronous cell growth, we also looked at the effect of FOXM1 and PLK1 inhibitors on BrdU incorporation when HPASMC were synchronized by withholding FBS in the culture medium followed by release into the cell cycle with serum addition. **Fig 2A** shows 0.2% FBS stimulated DNA synthesis in PAH cells. A considerably higher DNA synthesis took place at 5% FBS. Incubation with FOXM1 or PLK1 inhibitors prior to growth stimulation with FBS significantly reduced DNA synthesis at both 0.2% FBS and 5% FBS in the PAH HPASMC (**Fig 2A**). In the non-PAH cells low serum concentration (0.2%) did not promote DNA synthesis. At 5% FBS DNA synthesis took place in the non-PAH cells (**Fig 2B**). As in the PAH cells, preincubation with FOXM1 or PLK1 inhibitors significantly reduced the 5% FBS-stimulated DNA synthesis (**Fig 2B**).

### Expression of FOXM1 and PLK1 in HPASMC

**Fig 3:**
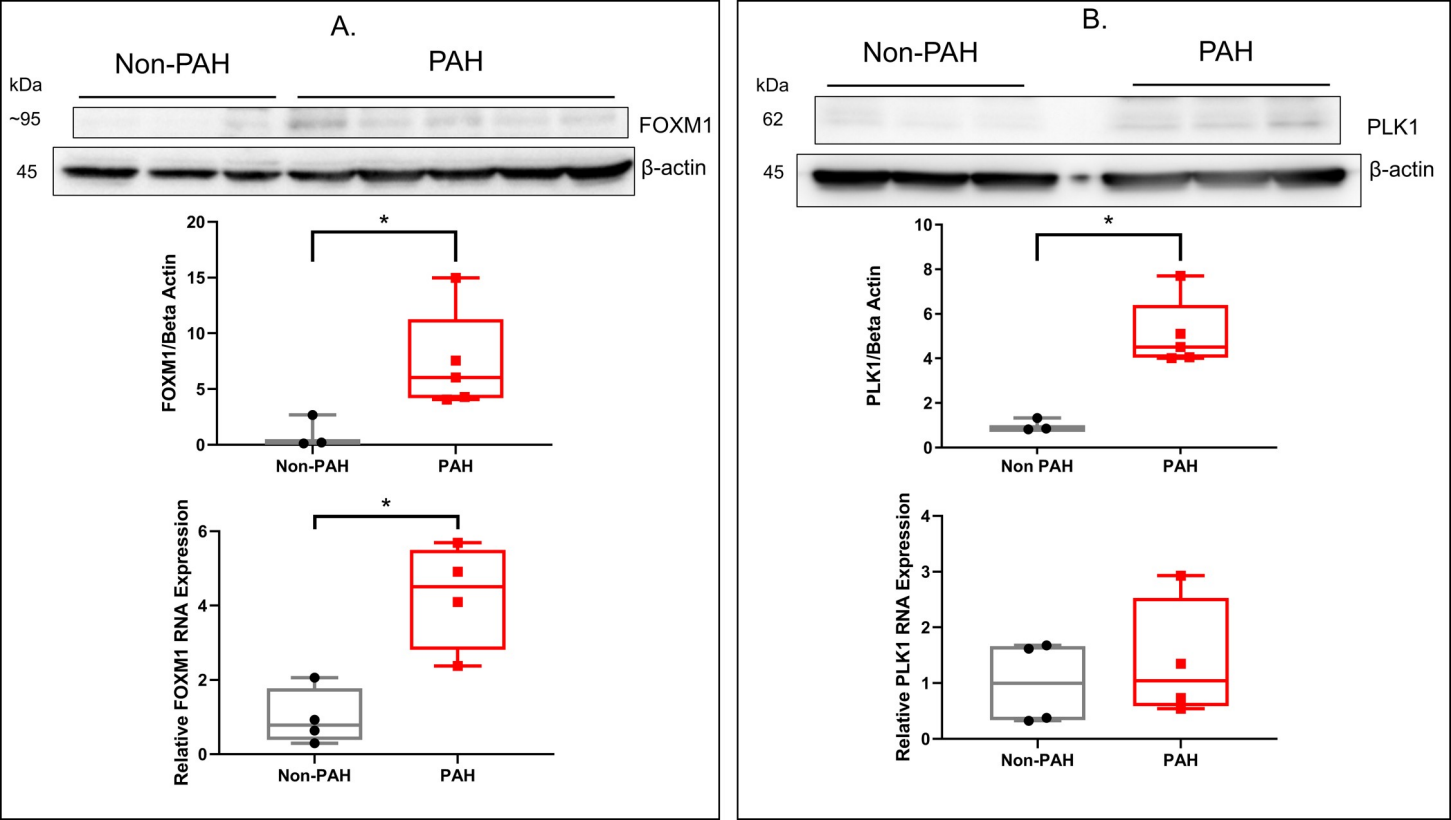
Induction of FOXM1 and PLK1 expression in non-PAH and PAH HPASMC. Representative western blots display the expression of FOXM1 (A) and PLK1 (B) generated by induction with 0.2% concentrations in non-PAH and PAH cells. The HPASMC were serum-starved for 24 hours and then treated with culture medium containing respective concentrations of FBS for another 24 h before protein isolation and western blot procedure. Box and whisker plots representing relative FOXM1 or PLK1 protein levels in n = 3 non-PAH and n = 5 PAH HPASMC. As discussed above, the blot intensity is relatively low due to the potency of available antibodies and the protein levels of PLK1 and FOXM1 particularly in the normal-HPASMC. Despite the low intensity, blot quantitation proved reproducible and these experiments were repeated at least three times. RNA expression (A, B) of *FOXM1* and *PLK1* in non-PAH and PAH cells after serum starvation and induction with 0.2% FBS containing medium for 7 h. RNA was determined with real-time qPCR. Box whisker plots show relative RNA values for n = 4 non-PAH and n = 4 PAH HPASMC. * = p < 0.05.

Clearly, both FOXM1 and PLK1 are involved in the regulation of proliferation of the HPASMC. Are these growth regulators more highly expressed in the PAH HPASMC than normal HPASMC? Protein levels of FOXM1 and PLK1 were determined in PAH and non-PAH cells under low and high FBS concentrations. Since non-PAH cells do not proliferate at 0.2% FBS while PAH cells do, we determined whether they have differential expression of FOXM1 and PLK1 at this low FBS concentration. **Fig 3A and 3B** illustrate protein levels of FOXM1 and PLK1 in normal and PAH HPASMC. The protein levels in PAH cells of both FOXM1 and PLK1 proved significantly greater at 0.2% FBS than normal cells at approximately 7 and 5-fold, respectively (**Fig 3A and 3B**). Given the faintness of the bands, considerable measures were taken to ensure proper band specificity for FOXM1 and PLK1 antibodies. These measures are illustrated in **S1**and **S2 Figs**. This was followed by comparison of normal and PAH HPASMC RNA expression using real time qPCR parallels the protein level results. Indeed, *FOXM1* RNA levels increased considerably in the PAH cells relative to those in normal-HPASMC as illustrated in **Fig 3B**. However, *PLK1* RNA was not significantly changed when normal and PAH HPASMC were compared (**Fig 3B**).

**Fig 4:**
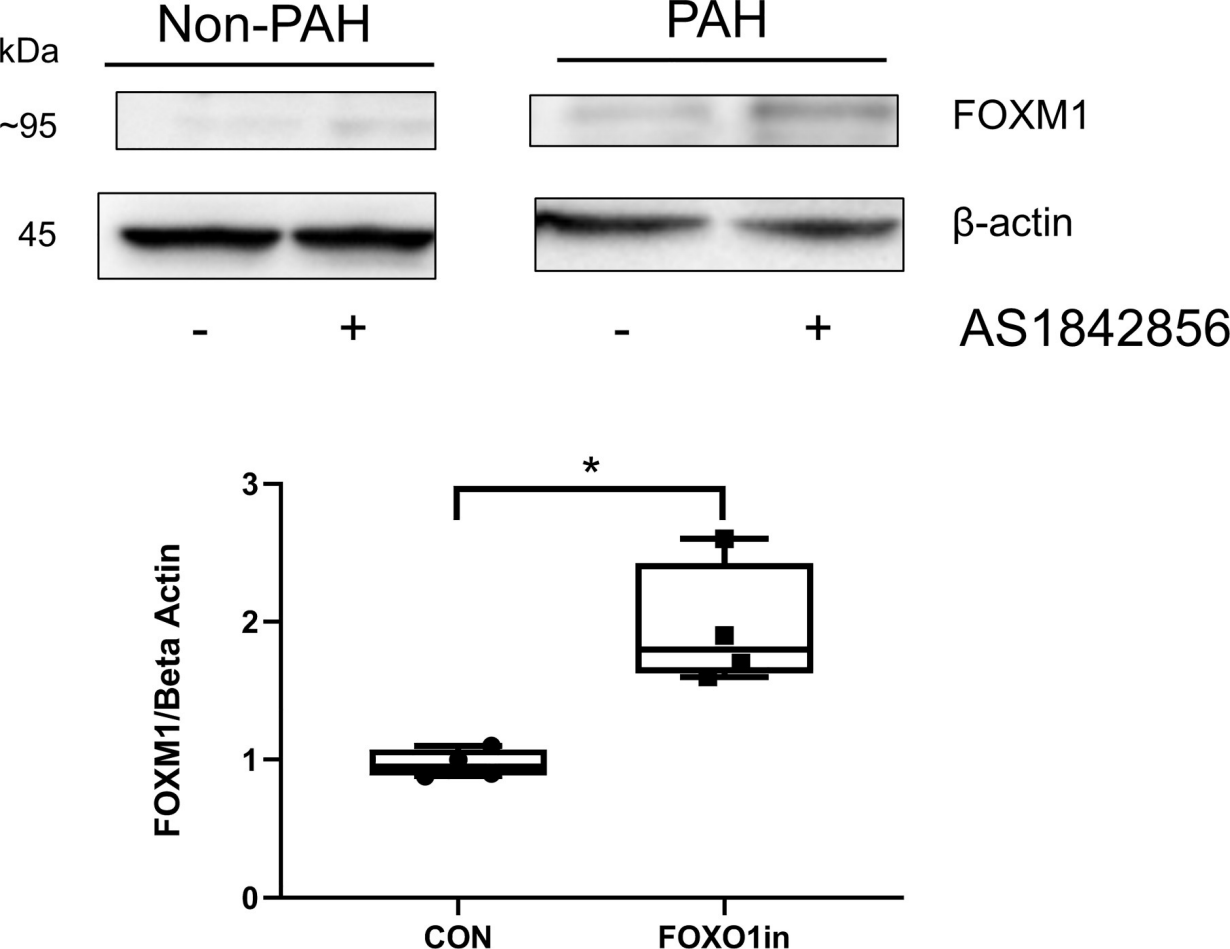
Effect of FOXO1 inhibition on FOXM1 expression. Cells were grown in medium containing 5% FBS for 24 h in the presence or absence of 1uM AS1842856 (FOXO1 inhibitor). Western blots were probed for FOXM1 and beta actin. Shown is a representative blot of a non-PAH and PAH HPASMC cropped from the same blot (uncropped image in **S3 Fig**). This experiment was done in duplicate. Box whisker plot displays the sum results of n = 4 HPASMC (two non-PAH and two PAH) treated with or without (CON) FOXO1 inhibitor. * = p < 0.05.

We then investigated, briefly, the relationship between FOXM1 and FOXO1 in the growth promoting FOXM1/PLK1 regulatory axis of the HPASMC. The activity of FOXO1 was inhibited using the pharmacological inhibitor, AS1842856, in the HPASMC. The action of FOXO1 on FOXM1 expression was then determined. The western blot in **Fig 4**illustrates that indeed a relationship exists. Inhibiting FOXO1 action induced about two-fold increase in FOXM1 expression in both non-PAH and PAH HPASMC.

### Expression and regulation of the cell cycle modulator, p27, by FOXM1 and PLK1

**Fig 5:**
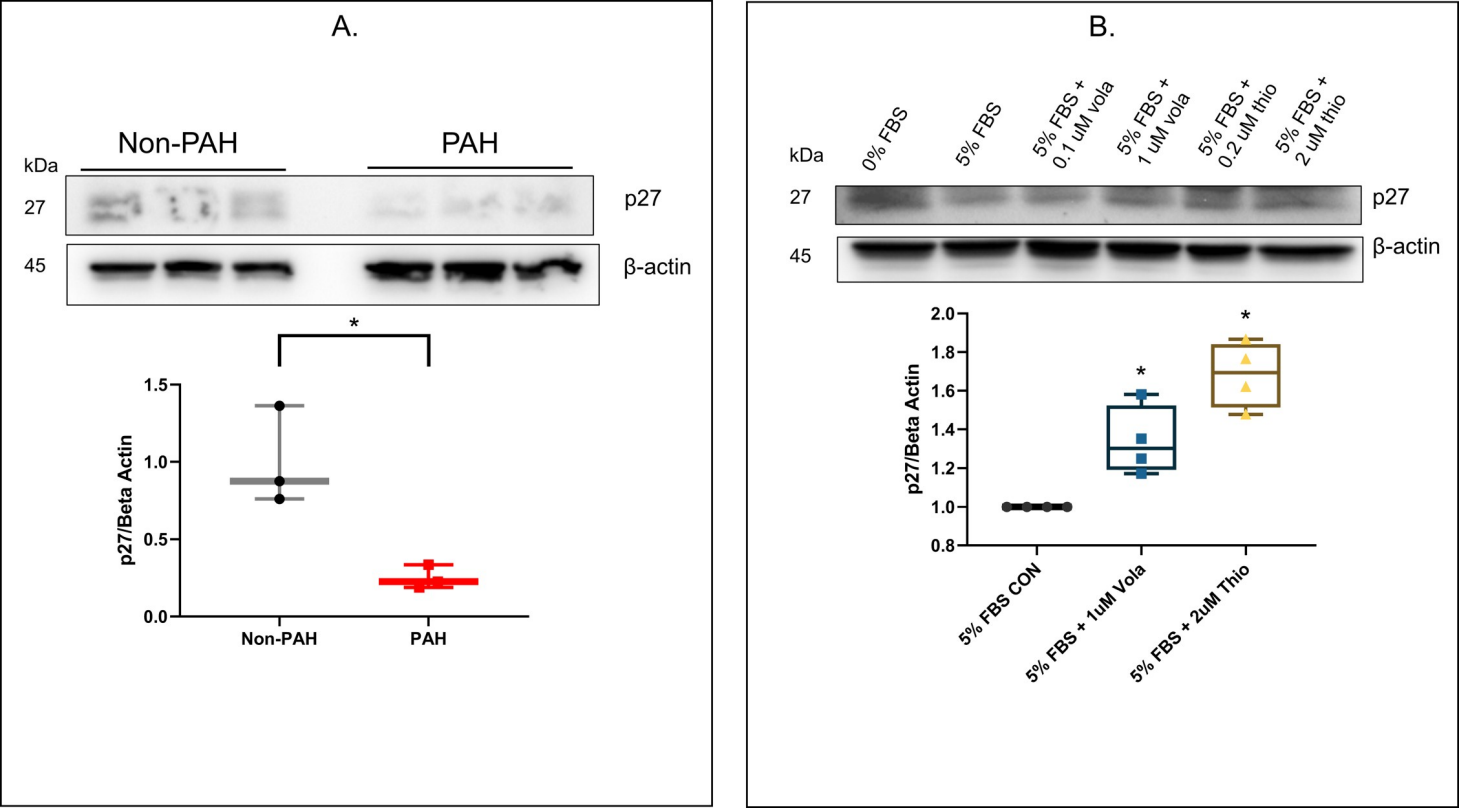
Effect of FOXM1 and PLK1 inhibition on p27 expression in PAH. (A) Representative western blot showing comparison of p27 expression in non-PAH vs. PAH HPASMC. Box whisker plots show the sum of results for n = 3 for non-PAH and PAH HPASMC. (B) HPASMC were serum starved for 24 h and pretreated in the presence or absence of either PLK1 inhibitor, volasertib (0.1 or 1 uM), or FOXM1 inhibitor, thiostrepton (0.2 or 2 uM) before addition of 5% FBS or vehicle (CON) for 24 h. Representative western blot of these results from one PAH HPASMC is shown. Box whisker plots show the sum of results for n = 4 PAH HPASMC treated with 5% FBS with or without (CON) 1 uM volasertib or 2 uM thiostrepton. These experiments were repeated three times. * = p < 0.05 vs 5% FBS.

p27 is an important regulator of the cycle progression in vascular smooth muscle cells [41]. It is part of the Cip/Kip family of cyclin dependent kinase (CDK) inhibitors. These inhibitors function by binding to an N-terminal kinase domain to alter the ATP binding pocket of the kinase and stop or slow down cell division cycle within the G1 and S phases. The effect of limiting FOXM1 and PLK1 activity on this cell cycle regulator was determined in PAH HPASMC. The cellular level of the p27 protein was reduced considerably in PAH HPASMC as illustrated in **Fig 5A.** Importantly, inhibiting the actions of either PLK1 or FOXM1 increased the expression of the p27 protein **(Fig 5B).**

### Regulatory interactions between FOXM1 and PLK1 expression in PAH HPASMC

**Fig 6:**
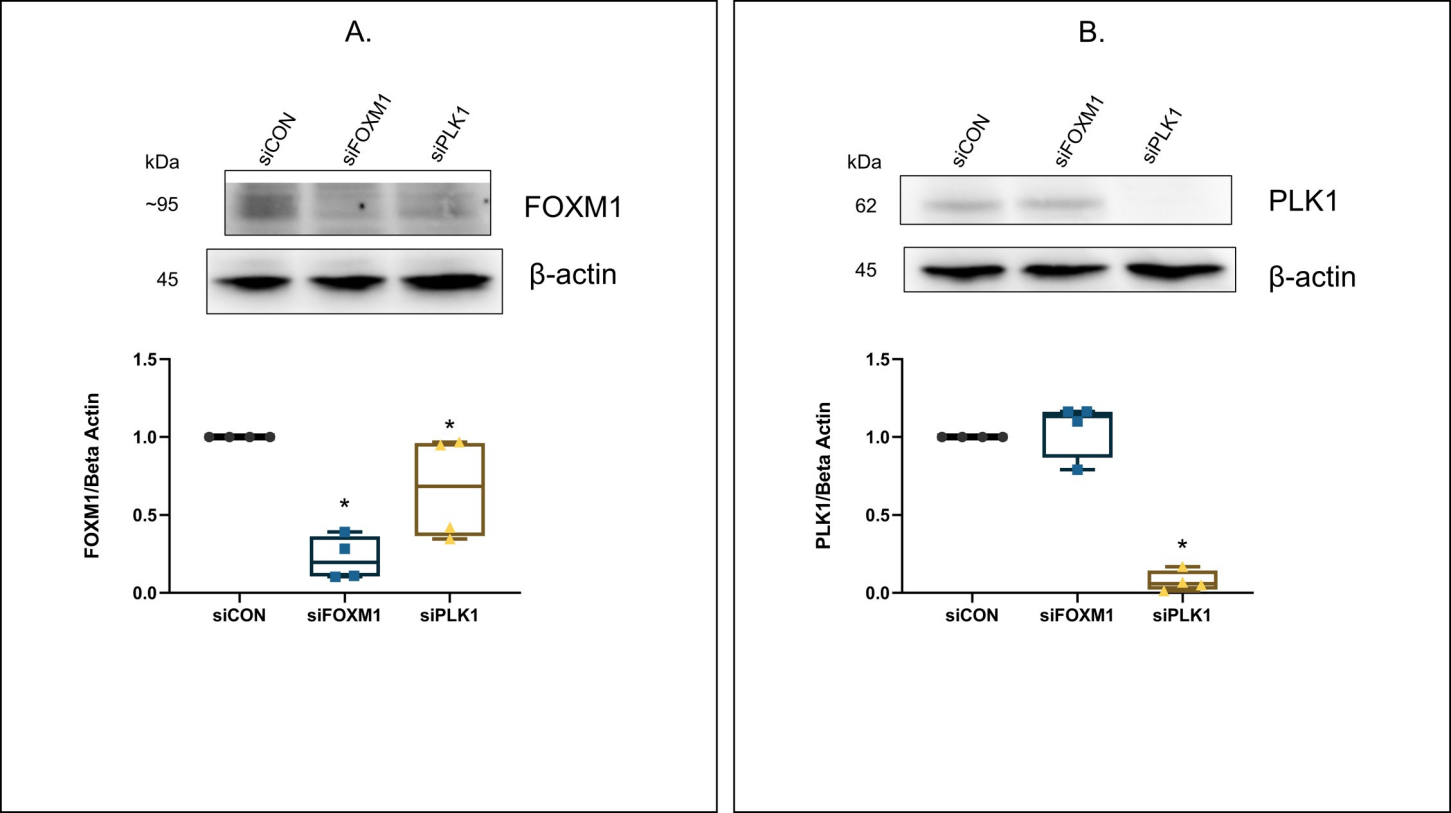
Effect of FOXM1 and PLK1 siRNA knockdown on FOXM1 and PLK1 protein expression in PAH HPASMC. HPASMC were transfected with control, FOXM1 and PLK1 siRNAs. Following transfection, the cells were cultured in medium containing 5% FBS for a 24 h. Proteins were harvested and probed for FOXM1 (A) or PLK1 (B) using western blots. A representative western blot of FOXM1 and PLK1 knockdown is illustrated. Box whisker plots show sum results from n = 4 PAH HPASMC. * = p < 0.05 vs siCON.

We then examined the relationship between PLK1 and FOXM1 expression. Results are shown in **Fig 6.** Using siRNA vs FOXM1 resulted in a markedly (~78%) reduced FOXM1 protein level (**Fig 6A**). siRNA vs PLK1 expression also reduced FOXM1 level, although less (~33%). However, PLK1 protein levels were only decreased by siRNA vs PLK1 (~93%) and not vs FOXM1 (**Fig 6B**).

### Regulation of downstream cell cycle components via FOXM1 and PLK1 actions

**Fig 7:**
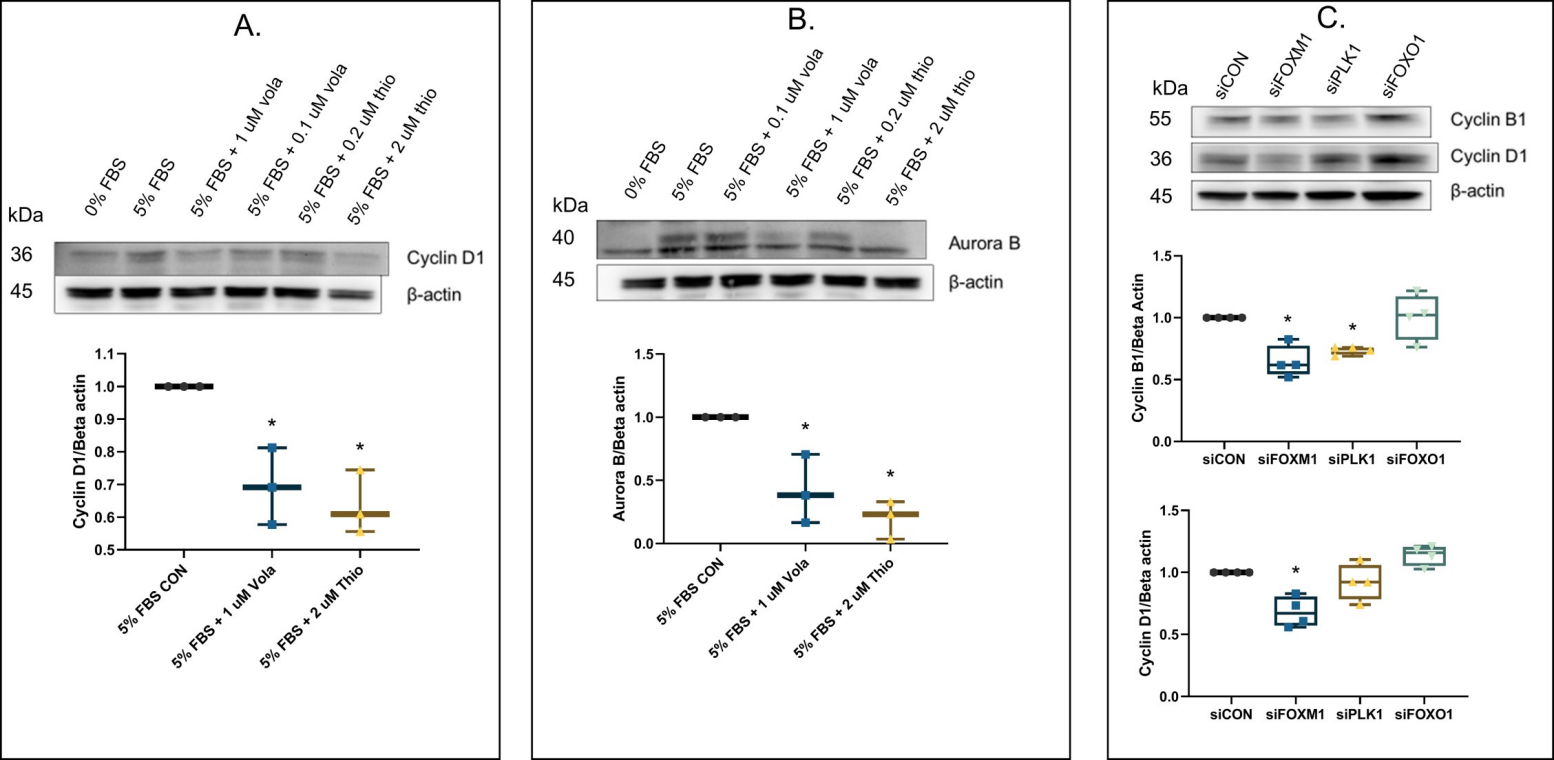
Effect of PLK1 and FOXM1 on protein expression of downstream components in PAH HPASMC. (A, B) HPASMC were serum starved for 24 h and pretreated in the presence or absence of either volasertib (0.1 or 1 uM), or FOXM1 inhibitor, thiostrepton (0.2 or 2 uM) before addition of 5% FBS or vehicle (CON) for 24 h. Proteins were harvested and probed for Aurora B (B) or cyclin D1 (A). A representative western blot image is shown. Box whisker plots show the sum of n = 3 PAH HPASMC in 5% FBS treated with or without 1 uM volasertib or 2 uM thiostrepton. * = p < 0.05 vs 5% FBS CON. (C) HPASMC were transfected with control, FOXM1 and PLK1 siRNAs. After transfection, cells were cultured in medium containing 5% FBS for a 24 h. Proteins were harvested and probed for cyclin B1 or cyclin D1 on western blots. A representative western blot image is shown. Box whisker plots show the sum of n = 4 PAH HPASMC in 5% FBS transfected with respective siRNAs. * = p < 0.05 vs siCON.

As illustrated, the expressions of PLK1 and FOXM1 are considerably elevated in PAH HPASMC. Also, the expression of these two growth factors is clearly interactive. Do these two proliferative agents in turn regulate the expression of downstream cell cycle components thus enabling more active proliferation? To answer this question, inhibiting the action of FOXM1 or PLK1 with chemical inhibitors strongly downregulated the expression of Aurora B and D1 as shown in **Fig 7A and 7B**. This was confirmed with use of siRNA vs PLK1 or FOXM1 which downregulated the expression of cyclin B1 and cyclin D1 (**Fig 7C**). siRNA vs FOXO1 had no effect on cyclin B1, but slightly elevated cyclin D1 (**Fig 7C**).

## Discussion

We previously used gene microarray to identify differentially expressed genes in HPASMC obtained from PAH patients compared to normal controls [7]. The genomic data provided an insight into the biology of PAH and supported what we and others have observed as hyperplastic growth of the PAH HPASMC [1, 3–5]. The data pointed to a heightened cell cycle progression as a primary area of altered gene expression based on the finding of increased expression of FOXM1 and PLK1. FOXM1 has previously been shown to participate in the etiology of experimental and clinical PAH [10–12]. However, little is known about the participation of PLK1. While inhibitors of PLK1 such as volasertib have been shown to reduce the proliferation of rapidly dividing tumor cells both *in vitro* and *in vivo* [42, 43], its possible role in PAH has received little attention. In addition, little is known of FOXM1 and PLK1 interactions and their participation in driving vascular cell growth in pulmonary hypertension.

In this study, we show that both FOXM1 and PLK1 participate in the hyperplastic growth of PAH HPASMC **(Fig 1)**.

Our cell growth determinations using both MTT and BrdU assays as well as siRNA transfections illustrate that when the action of either PLK1 or FOXM1 is blocked, not only is the proliferation of PAH HPASMC inhibited but also that of normal HPASMC (**Figs 1 and 2)**. Thus, these intracellular growth effectors activate proliferation under both normal and hyperplastic growth conditions. In conjunction with their proliferative phenotype, the PAH HPASMC express considerably higher protein levels of FOXM1 and PLK1 compared to normal HPASMC (**Fig 3**). Thus, FOXM1 and PLK1 are clearly participating in the highly accentuated PAH cell growth activity. However, it remains yet to be determined whether this is the primary trigger for the hyperplastic growth of PAH HPASMC. In fact, the PAH HPASMC are considerably more sensitive to FBS for growth than normal cells. These cells proliferate at a very low serum concentration (**Figs 1 and 2**) whereas normal cells do not, suggesting that other yet not understood upstream mechanism(s) are also participating in the hyperplastic behavior of these cells. Interestingly, we did not find an increase in *PLK1* RNA expression to match its protein increase observed in PAH cells. This could be due to PAH HPASMC upregulating PLK1 expression at the posttranscriptional/translational level or our time point was too short to capture the induction of the transcript. One report by Lee and coworkers showed that serum starved HEK293 cells stimulated with fetal bovine serum expressed *PLK1* RNA approximately 17 h later. This time point matched the induction of PLK1 protein and the cells entering G2/M phase [44]. It is likely our observation of elevated PLK1 and FOXM1 protein levels is the result of PAH HPASMC entering the cell cycle more readily at lower serum concentration.

Regardless, the increased expression of PLK1 in PAH cells is of great interest in PAH. PLK1 has been shown to play a critical role in cell mitosis [29, 45]. Recently, involvement of PLK1 has been reported in the hyperplasticity of blood vessels with intimal injury [46]. PLK1 has been reported to regulate the phosphorylation of FOXM1 and thus induce FOXM1 activity [29]. The siRNA and inhibitor experiments here illustrate further that PLK1 also regulates the protein expression of FOXM1 (**Fig 6**). The increased expression of PLK1 may well be an important aspect of PAH HPASMC hyperplasticity and goes in hand with reports of a decrease in FOXO1 (growth inhibitor) expression in PAH [28]. Which suggests that PLK1 and FOXM1 expressions are inversely related to the expression of FOXO1. Thus evidence in sum points to a corruption of regulated growth equilibrium in the PAH HPASMC.

A further indication of this altered control is the expression of the downstream component, p27. The level of p27, a known modulator of PASMC proliferation [41], is sizably reduced in the PAH cells (**Fig 5A**). Induction of p27 expression in mouse PASMC has been reported to counteract the stimulatory effects of hypoxia on cell proliferation. And administration of p27 to intact mice blunts hypoxic pulmonary hypertension [47]. Inhibiting the action of PLK1 increased the expression of p27 in PAH cells and inhibiting the action of FOXM1 brought levels of p27 back to those of non-dividing PAH cells (**Fig 5B**). Results in other biological systems have shown that FOXM1 promotes the degradation of p27 to allow cell growth to proceed [19].

Our results further indicate that the higher levels of FOXM1 and PLK1 found in PAH HPASMC induce increases in the expression of downstream components involved in the cell cycle such as Aurora B, cyclin B1 and cyclin D1. This is exemplified in **Fig 7A–7C** showing that both FOXM1 and PLK1 regulate this expression. This proliferative axis, including the reported lower levels of FOXO1 in PAH [28], forms at least a part of an overall hyperplastic activity of the PAH HPASMC.

In summary, results here demonstrate that PLK1 and FOXM1 are participants in the hyperplastic phenotype of PAH HPASMC and support the cancer paradigm for PAH vascular cells proposed by Rai and coworkers [48–51]. In fact, a report in the literature supports this idea, with complete reversal of severe idiopathic PAH following chemotherapy in a patient with a follicular B cell non-Hodgkin’s lymphoma [52]. Clearly, a hyperactive cell cycle progression appears to play an important role in the clinical phenotype of PAH. Our results are encouraging in highlighting a number of control points within the proliferative mechanism of PAH HPASMC that could be targeted to moderate or perhaps terminate the aberrant pulmonary vascular SMC hyperplasticity that characterizes cells derived from patients with IPAH.

**Fig 8:**
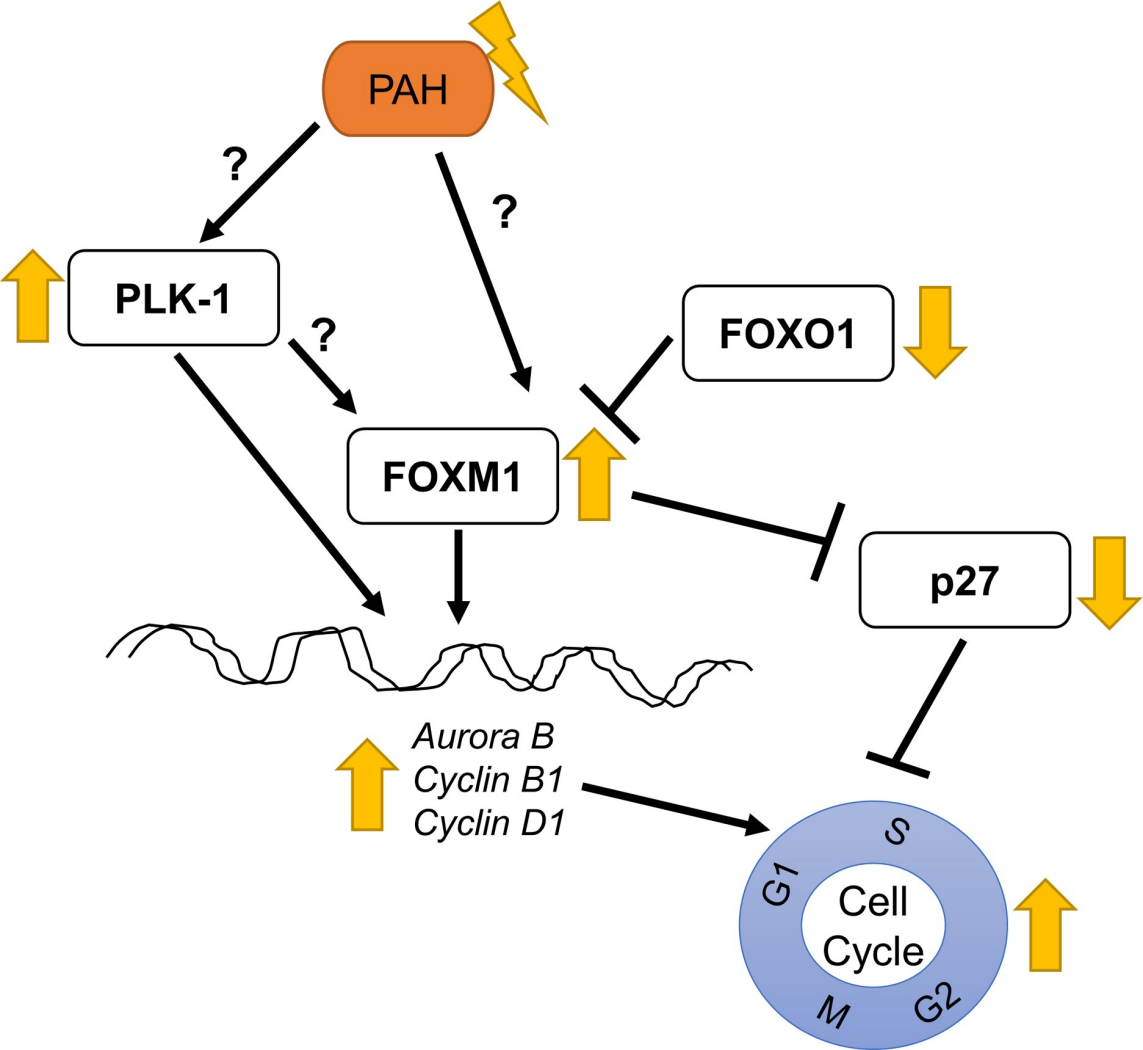
Schematic of PAH induced proliferation cascade in HPASMC. Our results suggest the following: PAH HPASMC exhibit upregulated presence of PLK1 and FOXM1. PLK1 then participates in the upregulated expression of FOXM1. The downregulation of FOXO1 activity in PAH further increases the presence of FOXM1. The upregulation of PLK1 and FOXM1 expression leads to the downregulated presence of the cell cycle inhibitor, p27. The overexpression of PLK1 and FOXM1 further leads to the overexpression of Aurora B, cyclin B1 and cyclin D1. The mechanisms responsible for this phenomenon in PAH are still under investigation. However, this process in sum may strongly contribute to the markedly increased cell cycle activity in PAH cells.

A diagram of the projected signaling cascade taking place in bringing about the accentuated growth of the PAH HPASMC is presented in **Fig 8.**

## Supporting information

S1 Fig Validation of FOXM1 antibody in IMR90 cells after 24 h treatment. IMR90 cells (human neonatal lung fibroblast cells) were incubated for 24 h in 0.2% FBS in DMEM medium before treatment for another 24 h with a change of medium of 0.2% FBS, 10% FBS or 10% FBS + 10 uM thiostrepton. Proteins were harvested and run on western blot. Blot was probed with antibodies against FOXM1 1:1000 (top image) and re-probed for beta actin 1:1000 (bottom image). Several non-specific FOXM1 bands ranging in size from 50 to 150 kDa are shown. However, a band at ~95 kDa is only visible in the cell’s growth state (10% FBS) and is abolished by thiostrepton (FOXM1 inhibitor) and therefore is the proper specific band for FOXM1 protein.
(PDF)

S2 Fig Representative full length blots for each antibody. (A) Full length blot probed for FOXM1 and beta actin showing correct band size. Blot shown from Fig 3. (B) Full length blot probed for PLK1 and beta actin showing correct band size. Blot shown from Fig 3. (C) Full length blot probed for p27. Blot shown from Fig 5. (D) Full length blot probed for Aurora B. Blot shown from Fig 7. (E) Full length blot probed with cyclin B1 (left) and re-probed with cyclin D1. Blot shown from Fig 7.
(PDF)

S3 Fig Uncropped blot image from Fig 4. Top image shows additional data showing AS1842856 (FOXO1 inhibitor) abolishing FOXO1 phosphorylation at Ser256 while also elevating the expression of FOXM1. Bottom image shows extra control treatment cropped out of Fig 4 image.
(PDF)

S1 Table List of HPASMC donor information. (DOCX)
