# Supplementary material for: Participation of PLK1 and FOXM1 in the hyperplastic proliferation of pulmonary artery smooth muscle cells in pulmonary arterial hypertension
Source: PLoS One. 2019 Aug 22;14(8):e0221728. doi: 10.1371/journal.pone.0221728 (PMC6705859; doi:10.1371/journal.pone.0221728)
Supplement: S1 Fig — IMR90 cells (human neonatal lung fibroblast cells) were incubated for 24 h in 0.2% FBS in DMEM medium before treatment for another 24 h with a change of medium of 0.2% FBS, 10% FBS or 10% FBS + 10 uM thiostrepton. Proteins were harvested and run on western blot. Blot was probed with antibodies against FOXM1 1:1000 (top image) and re-probed for beta actin 1:1000 (bottom image). Several non-specific FOXM1 bands ranging in size from 50 to 150 kDa are shown. However, a band at ~95 kDa is only visible in the cell’s growth state (10% FBS) and is abolished by thiostrepton (FOXM1 inhibitor) and therefore is the proper specific band for FOXM1 protein. (PDF) [file pone.0221728.s001.pdf]

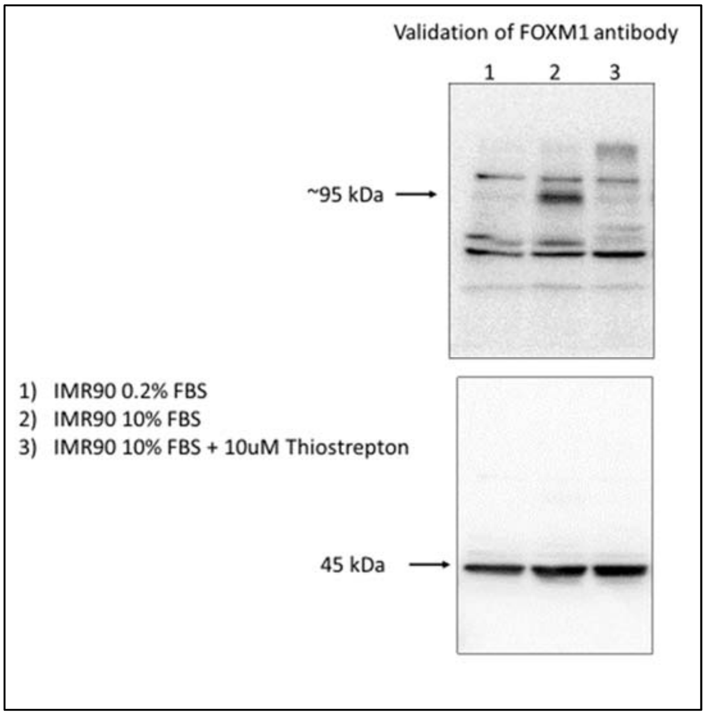

S1 Fig. Validation of FOXM1 antibody in IMR90 cells after 24 h treatment. IMR90 cells (human neonatal lung fibroblast cells) were incubated for 24 h in 0.2% FBS in DMEM medium before treatment for another 24 h with a change of medium of 0.2% FBS, 10% FBS or 10% FBS + 10 uM thiostrepton. Proteins were harvested and run on western blot. Blot was probed with antibodies against FOXM1 1:1000 (top image) and re-probed for beta actin 1:1000 (bottom image). Several non-specific FOXM1 bands ranging in size from 50 to 150 kDa are shown. However, a band at ~95 kDa is only visible in the cell's growth state (10% FBS) and is abolished by thiostrepton (FOXM1 inhibitor) and therefore is the proper specific band for FOXM1 protein.
