# Supplementary material for: Participation of PLK1 and FOXM1 in the hyperplastic proliferation of pulmonary artery smooth muscle cells in pulmonary arterial hypertension
Source: PLoS One. 2019 Aug 22;14(8):e0221728. doi: 10.1371/journal.pone.0221728 (PMC6705859; doi:10.1371/journal.pone.0221728)
Supplement: S2 Fig — (A) Full length blot probed for FOXM1 and beta actin showing correct band size. Blot shown from Fig 3. (B) Full length blot probed for PLK1 and beta actin showing correct band size. Blot shown from Fig 3. (C) Full length blot probed for p27. Blot shown from Fig 5. (D) Full length blot probed for Aurora B. Blot shown from Fig 7. (E) Full length blot probed with cyclin B1 (left) and re-probed with cyclin D1. Blot shown from Fig 7. (PDF) [file pone.0221728.s002.pdf]

A

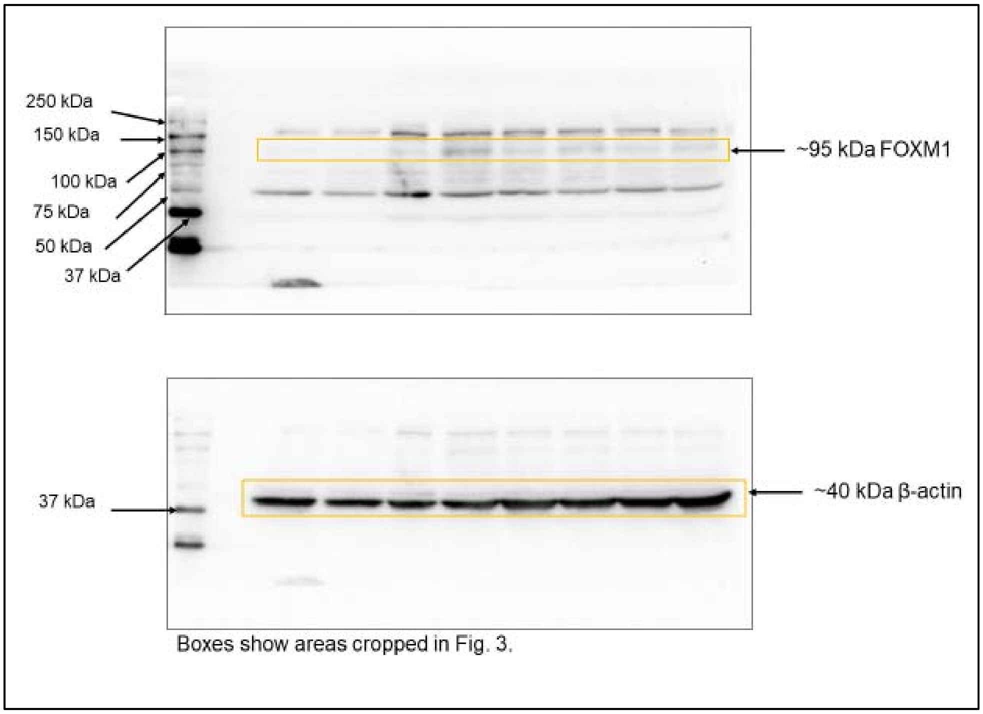
250 kDa
150 kDa
100 kDa
75 kDa
50 kDa
37 kDa
~95 kDa FOXM1
37 kDa
~40 kDa β-actin
Boxes show areas cropped in Fig. 3.

B

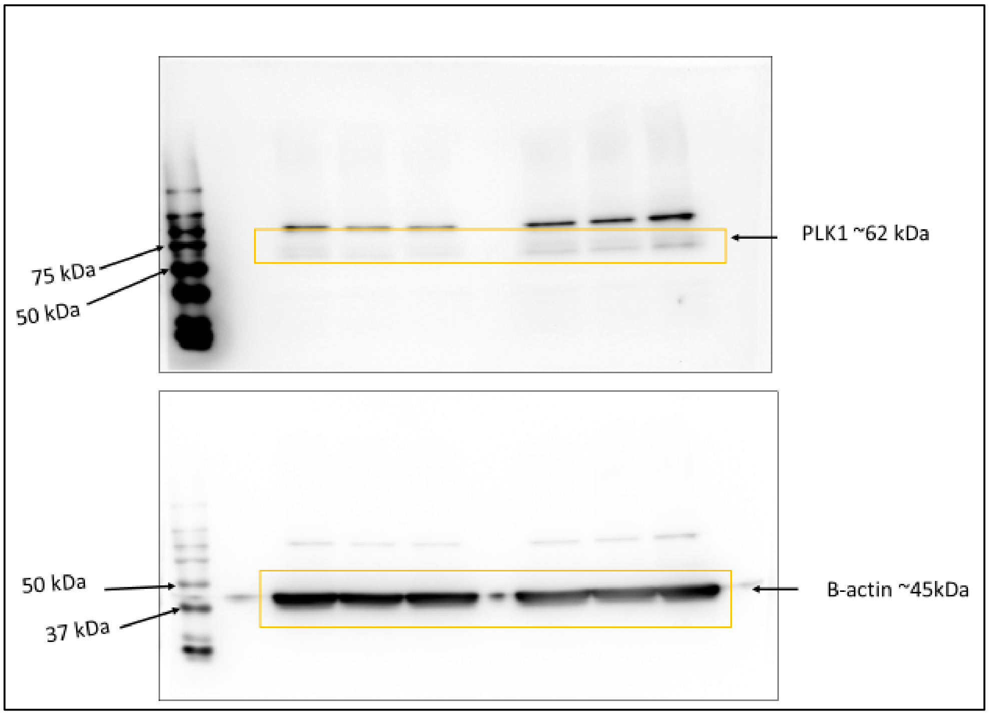
75 kDa
50 kDa
PLK1 ~62 kDa
50 kDa
37 kDa
B-actin ~45kDa

C

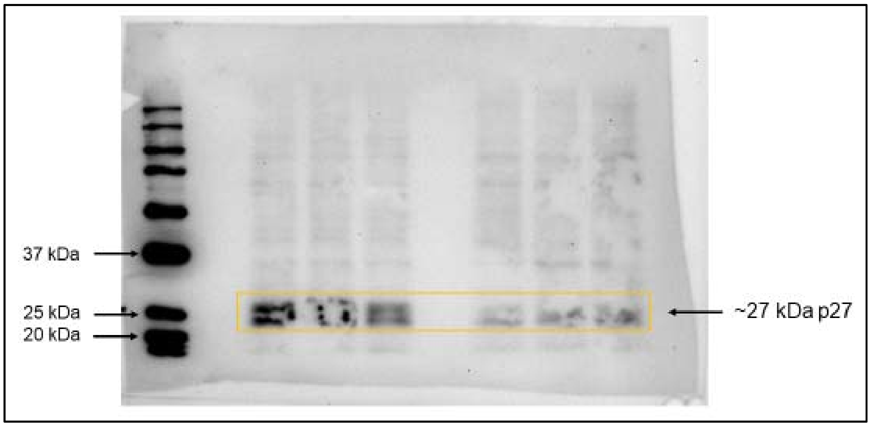
37 kDa
25 kDa
20 kDa
~27 kDa p27

D

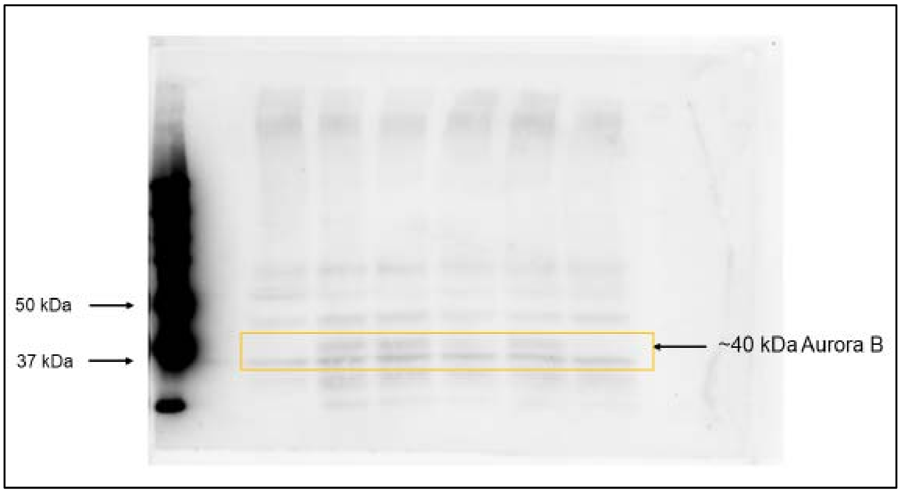
50 kDa
37 kDa
~40 kDa Aurora B

E

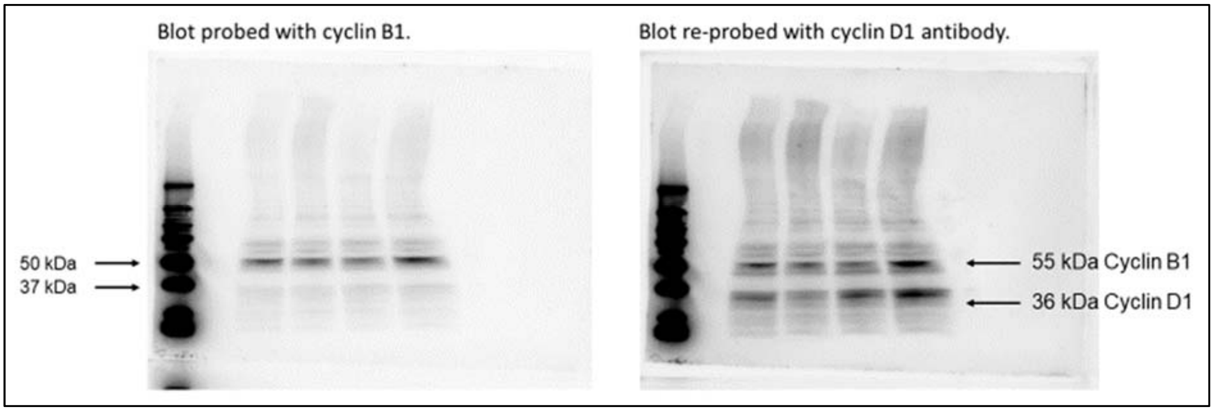
Blot probed with cyclin B1.
Blot re-probed with cyclin D1 antibody.
50 kDa
37 kDa
55 kDa Cyclin B1
36 kDa Cyclin D1

S2 Fig. Representative full length blots for each antibody. (A) Full length blot probed for FOXM1 and beta actin showing correct band size. Blot shown from Fig 3. (B) Full length blot probed for PLK1 and beta actin showing correct band size. Blot shown from Fig 3. (C) Full length blot probed for p27. Blot shown from Fig 5. (D) Full length blot probed for Aurora B. Blot shown from Fig 7. (E) Full length blot probed with cyclin B1 (left) and re-probed with cyclin D1. Blot shown from Fig 7.
