# Supplementary material for: Participation of PLK1 and FOXM1 in the hyperplastic proliferation of pulmonary artery smooth muscle cells in pulmonary arterial hypertension
Source: PLoS One. 2019 Aug 22;14(8):e0221728. doi: 10.1371/journal.pone.0221728 (PMC6705859; doi:10.1371/journal.pone.0221728)
Supplement: S3 Fig — Top image shows additional data showing AS1842856 (FOXO1 inhibitor) abolishing FOXO1 phosphorylation at Ser256 while also elevating the expression of FOXM1. Bottom image shows extra control treatment cropped out of Fig 4 image. (PDF) [file pone.0221728.s003.pdf]

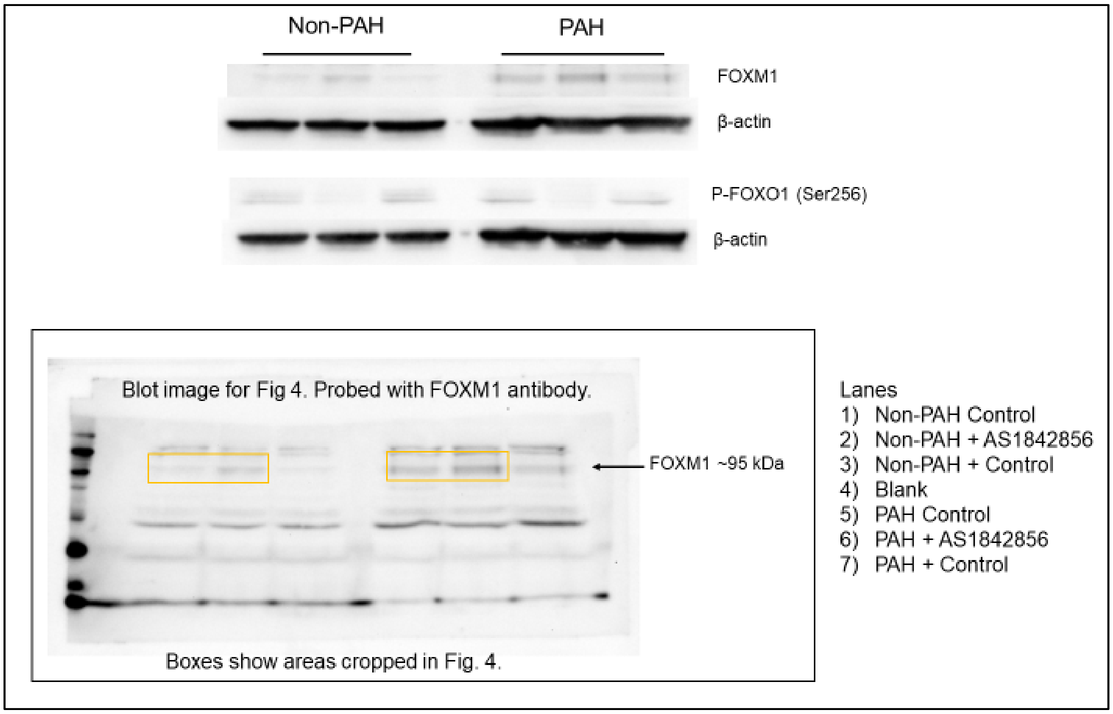

S3 Fig. Uncropped blot image from Fig 4. Top image shows additional data showing AS1842856 (FOXO1 inhibitor) abolishing FOXO1 phosphorylation at Ser256 while also elevating the expression of FOXM1. Bottom image shows extra control treatment cropped out of Fig 4 image.
