# Supplementary material for: Participation of PLK1 and FOXM1 in the hyperplastic proliferation of pulmonary artery smooth muscle cells in pulmonary arterial hypertension
Source: PLoS One. 2019 Aug 22;14(8):e0221728. doi: 10.1371/journal.pone.0221728 (PMC6705859; doi:10.1371/journal.pone.0221728)
Supplement: S1 Table — (DOCX) [file pone.0221728.s004.docx]

**S1 Table. List of HPASMC Donor Information**

| **Subject** | **Gender** | **Age** | **Germline Mutation** |
| --- | --- | --- | --- |
| **Donor Controls** |  |  |  |
| Control-1 | male | 36 | None |
| Control-2 | male | 39 | None |
| Control-3 | female | 48 | None |
| Control-4 | male | 43 | None |
| **Hereditary or Idiopathic PAH** |  |  |  |
| PAH-1 | male | 41 | BMPR2 del Exon 1-8 |
| PAH-2 | female | 26 | Smad-8 R294X |
| PAH-3 | male | 42 | None |
| PAH-4 | female | 34 | None |
| PAH-5 | Female | 47 | None |
